# Machine learning to improve the interpretation of intercalating dye-based quantitative PCR results

**DOI:** 10.1038/s41598-022-21010-z

**Published:** 2022-09-30

**Authors:** A. Godmer, J. Bigot, Q. Giai Gianetto, Y. Benzerara, N. Veziris, A. Aubry, J. Guitard, C. Hennequin

**Affiliations:** 1 Département de Bactériologie, AP-HP, APHP.Sorbonne Université, Hôpital Saint-Antoine, Paris, France; 2 INSERM, U1135, Centre d’Immunologie et des Maladies Infectieuses, Cimi-Paris, Sorbonne Université, Paris, France; 3 INSERM, Centre de Recherche Saint-Antoine, CRSA, AP-HP, Hôpital Saint-Antoine, Service de Parasitologie-Mycologie, Sorbonne Université, 75012 Paris, France; 4 Proteomics Platform, Mass Spectrometry for Biology Unit, UAR CNRS 2024, Institut Pasteur, Université de Paris, Paris, France; 5 Bioinformatics and Biostatistics HUB, Institut Pasteur, Université de Paris, Paris, France; 6 Laboratoire de Bactériologie-Hygiène, AP-HP, AP-HP.Sorbonne-Université, Hôpital Pitié-Salpêtrière, Paris, France

**Keywords:** Computational biology and bioinformatics, Molecular biology

## Abstract

This study aimed to evaluate the contribution of Machine Learning (ML) approach in the interpretation of intercalating dye-based quantitative PCR (IDqPCR) signals applied to the diagnosis of mucormycosis. The ML-based classification approach was applied to 734 results of IDqPCR categorized as positive (n = 74) or negative (n = 660) for mucormycosis after combining “visual reading” of the amplification and denaturation curves with clinical, radiological and microbiological criteria. Fourteen features were calculated to characterize the curves and injected in several pipelines including four ML-algorithms. An initial subset (n = 345) was used for the conception of classifiers. The classifier predictions were combined with majority voting to estimate performances of 48 meta-classifiers on an external dataset (n = 389). The visual reading returned 57 (7.7%), 568 (77.4%) and 109 (14.8%) positive, negative and doubtful results respectively. The Kappa coefficients of all the meta-classifiers were greater than 0.83 for the classification of IDqPCR results on the external dataset. Among these meta-classifiers, 6 exhibited Kappa coefficients at 1. The proposed ML-based approach allows a rigorous interpretation of IDqPCR curves, making the diagnosis of mucormycosis available for non-specialists in molecular diagnosis. A free online application was developed to classify IDqPCR from the raw data of the thermal cycler output (http://gepamy-sat.asso.st/).

## Introduction

PCR-based methods have emerged as essential tools for the diagnosis of infectious diseases. During the last decades, several refinements such as quantitative PCR either using specific probes or fluorescent intercalating dye, and more recently Lamp-PCR, have been proposed to optimize the detection of microbial DNA ^1^. Specific probes, even used in multiplex PCR, may uncover some rare species responsible for infection whereas multiplexing may reduce the sensitivity of the method ^2^. On the opposite, intercalating dye-based quantitative PCR (IDqPCR) enables the detection of larger groups of pathogens (at the level of genus, order or even phylum). This is counterbalances by the usual inability of these methods to specifically identify the pathogen even when the melting temperature (Tm) obtained after the denaturation of the amplicon can sometimes be used to distinguish between genera or species ^3–5^. Moreover, this method suffers some limitations such as the impossibility of multiplexing the PCR and the occurrence of a fluorescence signal resulting from non-specific DNA hybridization (typically primer dimers), as the dye can be incorporated into any form of double-stranded DNA. Thus, a careful analysis by experimented personal of the results is needed to limit the number of “doubtful result”.

We recently set-up such a method for the detection of Mucorales DNA in different specimens based on EvaGreen®, a fluorescent intercalating dye characterized by a low background fluorescence and almost no inhibitory effect in the PCR reaction^6,7^. This technique can detect in a single PCR 11 different Mucorales species belonging to 8 genera ^7^. However, between 10 and 15% of the results were considered doubtful, requiring additional investigation, typically other specimen to be tested. Machine learning (ML), a branch of artificial intelligence, focuses on the development of algorithms to learn from dataset in order to improve performances of their analysis regarding the solution to a stated problem based on the data they process. There are now a huge number of applications in medicine so-called computer-aided diagnosis, notably in the field of radiology, pathology and biomarkers. A possible application is to objectively classify specimens with questionable qPCR results, making this methodology an aid to interpreting results based solely on visual criteria.

The aim of this study was to evaluate the contribution of implementing a ML-based (ML) classification approach to the interpretation of the plots (amplification and denaturation curves) by comparing the performances of the “visual reading” and ML into IDqPCR results interpretation.

## Results

**Figure 1:**
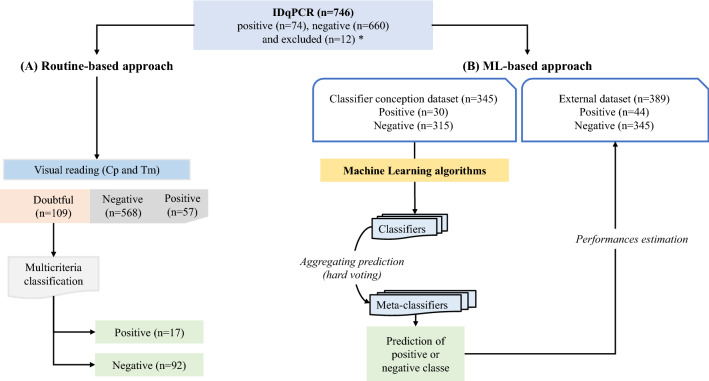
Design of the study for classification comparison of intercalating dye qPCR (IDqPCR) using two approaches (**A** and **B**). (**A**) “Routine based approach”: classification algorithm for intercalating dye qPCR (IDqPCR) based on the “visual reading” (with Tm for melting temperature and Cp for crossing point) and 2020 revised European Organization for Research and Treatment of Cancer and the Mycoses Study Group Education and Research Consortium (EORTC MSGRC) criteria^27^ and (**B**) “Machine Learning based approach”: classification based on ML algorithms classifier estimations. *Eleven IDqPCR were excluded from the study according due to the impossibility of rendering a final result with the current “Routine based approach” (lack of clinical data essential for diagnosis).

A total of 734 IDqPCR results were previously classified by “visual reading” according to both objective (Cp for Crossing point and Tm) and subjective (shape of the curves) characteristics of the amplification and denaturation curves. This returned 57 (7.7%), 568 (77.4%) and 109 (14.8%) positive, negative and doubtful results, respectively (Fig. 1A). The integration with multicriteria on doubtful results allowed to categorize them positive (n = 17) or negative (n = 92) (Supplementary Appendix 1). Despite this complementary analysis, 12 results cannot be assigned as positive or negative and were excluded from the analysis. All correctly labelled IDqPCR were then used to evaluate the “ML-based approach” (Fig. 1B). Two datasets were generated, the first named the classifier conception dataset (n = 345) was used to create classification based on ML-algorithms classifiers whose performances were evaluated on the remaining data named the external dataset (n = 389). Predictions of each classifier were aggregated to form a meta-classifier and to give final predictions on the external dataset.

### Performances of the “ML-based approach”

**Figure 2:**
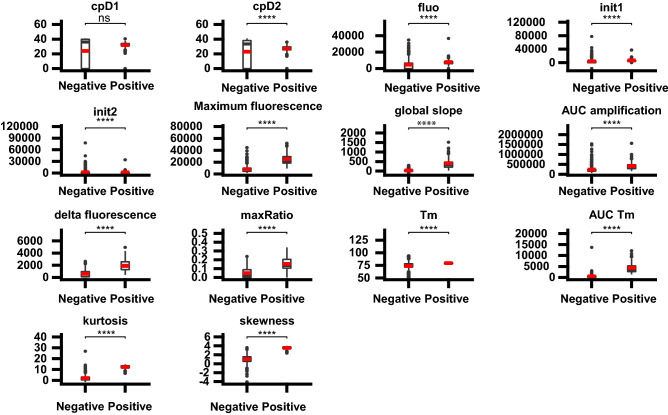
Boxplots of 14 features calculated from the intercalating dye-based quantitative PCR curves comparing positive and negative classes.* Notes*: The mean is represented by a red crossbar; means were compared using Wilcoxon rank sum test:****(significant difference with* p* value < 10–4 and ns (non significant difference with* p* value > 0.05). cpD1 (Cp at the first maximum derivative of the amplification curve); cpD2 (Cp at the second maximum derivative of the amplification curve); fluo (the fluorescence value the maximum of the second derivative curve (cpD2)); init1 (the initial template fluorescence from the sigmoidal model); init2 (the initial template fluorescence from an exponential model); maximum fluorescence (the maximum of fluorescence of the amplification curve); global slope (the slope of the amplification curve using a linear regression model); AUC amplification (Area Under the amplification Curve); delta fluorescence (the difference of fluorescence between the minim and the maximum of fluorescence); maxRatio (this method allows the identification of a coherent point in or very close to the exponential region of the qPCR signal). Tm (melting temperature), AUC Tm: area under the melting curve, kurtosis (measure of shape concerning the melting curve) and skewness (Skewness, measure of asymmetry of the melting curve).

Fourteen features were extracted from the raw data of amplification and melting curves (see Methods section). These features were the input data for the ML-algorithms. The mean value of each feature expects one (Cp at the maximum of the second derivative of the curve; *P* = 0.10) differed significantly between the positive and the negative classes (*P* < 0.05) (Fig. 2). All these features calculated from the classifier conception dataset (n = 345) were used for the “ML-based approach” which allowed the definition of 48 meta-classifiers further estimated on the external dataset (n = 389).

### Performances of the classifiers

**Figure 3:**
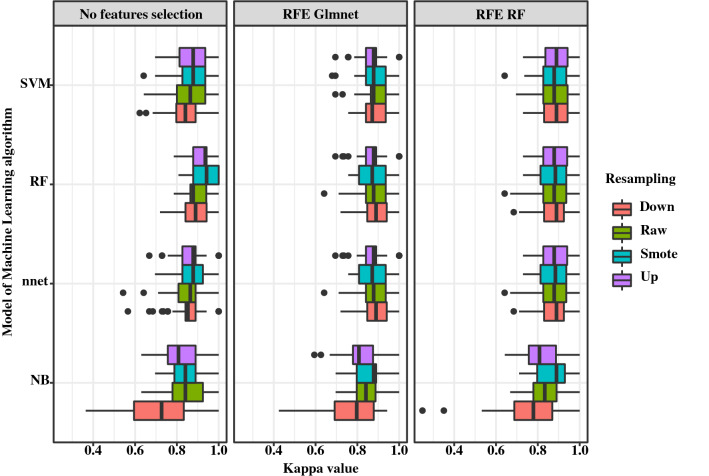
Comparison of Machine Learning algorithms, resampling and feature selection methods to estimate with the Kappa coefficient a classification model on test sets. Notes SVM for Linear Support Vector Machine, RF for Random Forests and, NB for Naive Bayes and nnet for single hidden layer Neural NETwork; Recursive Feature Elimination (RFE) coupled to Random forests (RF) or Logistic Regression (Glmnet) or No selection variable method; resampling methods (Up, Down or SMOTE ^8^) or no sampling method (raw).

Performances of the algorithms were assessed on Kappa coefficient and its standard deviation. The RF (Random Forests) algorithm without feature selection and combined with SMOTE (Synthetic Minority Sampling Technique) ^8^ or the up-sampling as resampling methods returned the best performances (mean Kappa = 0.93 ± 0.06 and 0.92 ± 0.06, respectively). In contrast, the NB (Naive Bayes) algorithm combined with the down-sampling method resulted in lower performances on the test set (mean Kappa = 0.76 ± 0.14, 0.78 ± 0.12 and 0.71 ± 0.16) (Fig. 3 and Supplementary Table S1). The three ML-algorithms (SVM, RF and nnet) gave similar performances whatever the resampling method and the feature selection method. In contrast, the NB algorithm returned lower performances with less accurate predictions, notably when using the down resampling method. This suggests all ML algorithms except NB can be used confidently to classify results as positive or negative with data from the classifier conception dataset.

### Performances of the meta-classifiers on the external dataset

Accuracies, Kappa coefficients and F1-scores were greater than 0.97, 0.83 and 0.98 respectively for all meta-classifiers. Nevertheless, among the 48 meta-classifiers, 42 were limited in their performances notably the specificity (n = 33), the sensitivity (n = 6), or both (n = 3) (Supplementary Table S2). Considering independently the ML-algorithms, the resampling and the selection feature methods, the highest overall performances of the different meta-classifiers were obtained with the NB algorithm (mean Kappa = 0.99 ± 0.01), the down-sampling (mean Kappa = 0.98 ± 0.02) and the RFE-Glmnet (Recursive-Feature-Elimination selection coupled to logistic regression) selection feature methods (mean Kappa = 0.96 ± 0.04) (Supplementary Table S3). Six of the 48 meta-classifiers returned a total agreement on the external dataset (mean Kappa = 1). The 6 meta-classifiers were obtained with the following combinations (ML-algorithm/Feature-selection-method/Resampling-method): NB/RFE-Glmnet/Down, NB/RFE-Glmnet/SMOTE, NB/no-selection/no-resampling, NB/RFE-RF/SMOTE, RF/no-selection/Down and RF/RFE-RF/Down.

## Discussion

IDqPCR is an easy-to-implement and cost-effective technique for the detection of microbial DNA in microbiology labs. It only requires the proper design of 2 primers that would be able to amplify species, genus or even higher taxonomic ranks in a single PCR assay. However, as the dye incorporates in any kind of double strand DNA, multiplexing is impossible on one side, while on the other side, the method is subject to non-specific fluorescence signals due to non-specific hybridizations ^9^. Therefore, only looking at the amplification and denaturation plots may not lead to the definitive results in some cases (14.8% in our study). Here, we investigated the usefulness of a “ML-based approach” applied to IDqPCR results to improve the certainty of diagnosis. Indeed, supervised ML is an appealing artificial intelligence method to classify biological results into known categories whose help has been proven in different health diagnosis contexts ^10–12^.

In order to apply this approach to the amplification and denaturation curves, we characterized the behaviour of curves with several key features such as the maxRatio value for the amplification curve. This feature had already been used for building ML models based on SVM algorithms, returning an accuracy at 1 for high-throughput qPCR analysis classification ^13^. The asymmetry (skewness) and the distribution of fluorescence asymmetry (kurtosis) were the most informative features comparing to the Tm and AUC Tm of denaturation curves. Moreover, differences in the mean value of these features between positive *versus* negative classes were the most significant (*P* < 2.10^–44^) compared to other selected features (Supplementary Table S4). Moreover, these two features seem to be important to the classifiers because they were rarely discarded by the two feature selection methods used (Supplementary Table S5).

Due to the low prevalence/incidence of mucormycosis in the studied population (74 positive results among 734 samples), the datasets were imbalanced. This is a commonly encountered characteristics in medical contexts and represents a challenge for ML techniques ^14–17^. Thus, different known approaches were used in this study such as resampling the data before training the algorithms, eliminating some non-informative features with feature selection methods or combining the performance of several classifiers ^14,18^. We used popular ML-algorithms to solve classification issues trained with the Kappa metric commonly used for imbalanced data ^19–22^.

Applied to the classifier dataset, the best result was obtained with the RF algorithm either combined with no-feature selection or the SMOTE method (mean kappa at 0.93 ± 0.06). However, all algorithms used for classifiers conception returned Kappa values higher than 0.83, these algorithms could provide interesting results on other datasets. In order to provide robust results, we used meta-classifiers consisting of creating different classifiers estimated on different training sets and aggregating their predictions with the hard voting method. This strategy has already been successfully applied in medical biology on more complex data such as microarray data ^23^. On our external dataset, 6 meta-classifiers out of 48 returned perfect Kappa values: 4 and 2 were obtained with NB and RF algorithms respectively, both combined to the down-sampling method. Interestingly, the mean Kappa values for all ML algorithms, and the NB-based meta-classifiers gave the best mean Kappa (0.99 ± 0.01) on the external dataset whatever the feature selection method or the resampling method. Yet, the NB classifiers were the ML-algorithms with the lowest mean and the highest standard deviation of Kappa values on the classifier dataset. The better performance of the NB-based meta-classifiers can be explained by the fact that the classifiers included returned predictions less correlated to each other one than the other ML algorithms. Although these NB classifiers provide a worse estimation taken independently to each other, using the hard voting method improves the classification results. Conversely, other classifiers giving correlated predictions may all be wrong at the same time, not allowing the results to be improved as much as NB classifiers when used in meta-classification.

Mucormycosis is a life-threatening invasive fungal disease whose successful treatment relies on an early and reliable diagnosis ^24^. Doubtful IDqPCR results requires the expertise of a molecular biology specialist contrary to the “ML-based approach”.

In order to further facilitate the interpretation of those results, we implemented the 6 meta-classifiers providing perfect predictions on the external dataset in a free online application using the raw data from amplification and denaturation plots to return a positive or a negative result (http://gepamy-sat.asso.st/).

## Methods

### Mucormycosis case definition

From January 2019 to December 2021, a total of 746 intercalating dye qPCR (IDqPCR) were performed according to a previously published protocol ^7^. Results were classified as positive, negative or doubtful according to a “visual reading” of the amplification curve (exponential increase in the fluorescence index) and denaturation curve (shape of a peak). In addition, to be considered positive, a specimen should have a crossing point (Cp) < 40 in the amplification curve, and a Tm between 77 and 82 °C, limits based on temperatures obtained with DNA extracted from 57 strains representative of 8 Mucorales genera and 11 species. Specimens with a Cp > 40 or a Cp < 40 and a Tm out of 77–82 °C range were considered negative. In the case of a Cp < 40 with a Tm between 77–82 °C but with an atypical peak on the melting curve, such as multi-peaks or flattened peak, the result of the IDqPCR was categorized as doubtful. All the results were then introduced in a multicriteria classification (clinical, radiological and microbiological) based on the 2020 revised European Organization for Research and Treatment of Cancer and the Mycoses Study Group Education and Research Consortium (EORTC-MSGRC). In addition, we used the outcome under treatment to make a final classification for the diagnosis of mucormycosis. In the light of this analysis, doubtful results of IDqPCR were classified for this study as positive or negative ^25^. This strategy was called “routine-based approach”.

### Machine learning study design

The whole dataset of 746 IDqPCR was splitted in 2 according to the study period. Data from 2019–2020 were used to create the classifier conception dataset dedicated to the definition of different classifiers based on ML-algorithms, while data from 2021 formed the external dataset used to evaluate the classifiers performances**.** After integrating the multicriteria analysis, the classifier conception dataset included a total of 345 IDqPCR results labelled positive (n = 30) and negative (n = 315). The external dataset included 401 IDqPCR results labelled positive (n = 44) or negative (n = 345). Twelve samples were excluded due to the impossibility of rendering a result with the “routine-based approach” (Supplementary Appendix 1). The “ML-based approach” corresponding to the predictions from classifiers (predicted positive or negative class) on the external dataset were compared to the results from the “routine-based approach” (Fig. 1).

### Selected features from the amplification and denaturation plots

Several features were calculated from the complete raw dataset of fluorescence from the two amplification and denaturation curves types using three R packages ^26–28^ .

From the amplification curve, 10 features were retained: the Cp of the maximum first derivative of the curve (CpD1); the Cp at the maximum of the second derivative of the curve (CpD2); the initial template fluorescence from the sigmoidal model (init1); the initial template fluorescence from an exponential model (init2); the fluorescence value of the maximum of the second derivative curve (fluo); the maximum of fluorescence of the curve (maximum fluorescence); the slope of the curve using a linear regression model (global slope); the Area Under the Curve (AUC amplification); the difference between the minimum and the maximum of fluorescence (delta fluorescence) and the value of the maxRatio method which allows the identification of the beginning of the exponential region of the qPCR signal (maxRatio) ^29^.

From the melting curve, 4 features were retained: the Tm, the Area Under the curve (AUC Tm); the kurtosis that measures of the "tailedness" of the peak and the skewness measuring the asymmetry of the curve. Means of each feature from positive *versus* negative classes were compared using the Wilcoxon rank sum test, using Benjamini–Hochberg adjustment. The P threshold was fixed at 0.05 for statistical significance.

### Machine learning analysis

**Figure 4:**
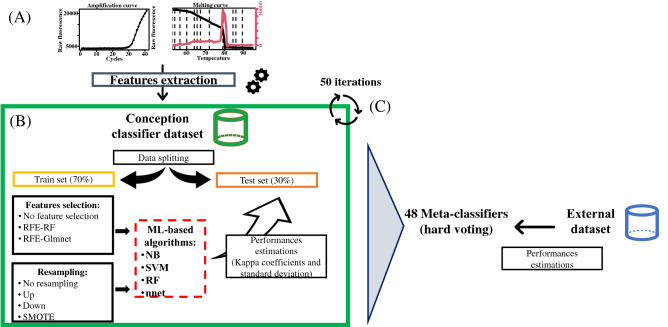
Pipelines for classifiers development and estimation of performances with the external dataset in three steps (**A**, **B** and **C**). (**A**) Extraction 14 features from amplification and melting curves using the R packages ^25–27^; splitting the conception classifier dataset (345 IDqPCR) into train and test datasets (**B**) Conception of classifiers (NB for Naive Bayes, SVM for Linear Support Vector Machine, RF for Random Forests and nnet for single-hidden-layer Neural NETwork with several pipelines: (i) selection of features using Recursive Feature Elimination (RFE) coupled to Random forests (RF) or Logistic Regression (Glmnet) or without selection variable method. (ii) different resampling methods (Up, Down or SMOTE ^30^) or no resampling method. The steps (**A**) and (**B**) were repeated with 50 iterations using a random loop and generated 2400 classifiers (**C**) Performances of 48 meta-classifiers grouped by Machine Learning algorithm, resampling, selection variable methods and based on hard voting method were estimated on the external dataset (389 IDqPCR).

Several ML-based algorithms using raw data from amplification and melting curves were created to categorize IDqPCR results into a positive or negative class using different pipelines built with the R caret package (Fig. 4) ^30^.

Fourteen features were extracted from amplification and denaturation curves and their mean calculated for both classes (Fig. 4A). Next, algorithms also called classifiers were applied to the classifier conception dataset with a random loop with 50 iterations to generate different train sets (70% of the data for training classifiers) and test sets (30% of the data for performance estimation of each classifier) (Fig. 4A).

Several pipelines were tested in order to create various classifiers (Fig. 4B). Two methods were tested to discard non-informative features: Recursive-Feature-Elimination coupled to random forests (RFE-RF) or to logistic regression (RFE-Glmnet) with k-folds cross validation (k = 5) (Fig. 4B). This procedure consists in dividing the dataset into k-folds (k = 5). In the first iteration, the first fold is applied to test the algorithm while the others are used to train it; this process is repeated until each fold has been used as a test set.

Because the datasets had an imbalanced ratio ≥ 3, meaning they are at least medium-imbalanced, we tested both raw imbalanced data and new-generated re-balanced data (Supplementary Appendix  2). Re-balanced data were generated using 3 resampling methods: down-sampling randomly removing instances in the majority class (negative IDqPCR class), up-sampling randomly replicating instances in the minority class (positive IDqPCR class) and Synthetic Minority Sampling TEchnique (SMOTE), synthesizing new minority instances using a ML-algorithm (K-nearest neighbors) (Fig. 2B) ^8^. The classifiers were previously trained with or without a feature selection and/or resampling methods.

Four different ML-algorithms were implemented: Random forests (RF), linear Support Vector Machine (SVM), single-hidden-layer Neural NETwork (nnet) and Naive Bayes (NB). All classifiers were trained with k-folds cross validation (k = 5). The best hyperparameters whose values needed to be adjusted for the learning algorithms were estimated using a specific search grid or a random search grid (Supplementary Table S6). The ML-algorithms were trained with the Cohen's Kappa coefficient metric which allows to assess the inter-rater reliability that varies from − 1 (total disagreement) to 1 (total agreement) ^19^. The mean Kappa coefficient and standard deviation (sd) of each classifier on the test set were used to evaluate the more reliable classifiers ensemble before estimating their performances on the external dataset.

Then, the predictions from each classifier (including the ML-algorithm, resampling and selection features methods) were aggregated with the majority rule voting (hard voting) into a meta-classifier for the final prediction (Fig. 4C). A total of 48 meta-classifiers were generated by crossing feature selection, resampling methods and ML-algorithms.

### ML performances estimation

The performances of these meta-classifiers were estimated on the external dataset (Fig. 4C) using accuracy (number of correctly predicted data), Negative Predictive Value (NPV) (proportion of the negatives cases giving negative results), Predictive Positive Value (PPV) (proportion of the positives cases giving positive results), sensitivity (true-positive recognition rate)**,** specificity (true-negative recognition rate), F1-score (harmonic mean of PPV and sensitivity) and the Kappa coefficient (Supplementary Appendix 3).

## Supplementary Information

Supplementary Information.

## Data Availability

Datasets used and/or analyzed during the current study are available from the corresponding author upon reasonable request.
