# Supplementary material for: Machine learning to improve the interpretation of intercalating dye-based quantitative PCR results
Source: Sci Rep. 2022 Sep 30;12:16445. doi: 10.1038/s41598-022-21010-z (PMC9525288; doi:10.1038/s41598-022-21010-z)
Supplement: Supplementary file 1 — Supplementary Information. [file 41598_2022_21010_MOESM1_ESM.pdf]

<u>**Supplementary Material**</u>

Machine Learning to improve the interpretation of intercalating dye-based quantitative PCR results

A. Godmer[1,2], J. Bigot[3], Q. Giai Gianetto[4,5], Y. Benzerara[1], N. Veziris[1,2], A. Aubry[2,6], J. Guitard[3], C. Hennequin[3]

[1]AP-HP, APHP.Sorbonne Université, Hôpital Saint-Antoine, Département de Bactériologie, Paris, France

[2]Sorbonne Université, INSERM, U1135, Centre d'Immunologie et des Maladies Infectieuses, Cimi-Paris, Paris, France

[3]Sorbonne Université, INSERM, Centre de Recherche Saint-Antoine, CRSA, AP-HP, Hôpital Saint-Antoine, Service de Parasitologie-Mycologie, F-75012 Paris, France

[4]Institut Pasteur, Université de Paris, Proteomics Platform, Mass Spectrometry for Biology Unit, UAR CNRS 2024

[5]Institut Pasteur, Université de Paris, Bioinformatics and Biostatistics HUB

[6]AP-HP, AP-HP.Sorbonne-Université, Hôpital Pitié-Salpêtrière, Laboratoire de Bactériologie-Hygiène, Paris, France

Index

Supplementary material Appendix 1: Interpretation of the doubtful results of IDqPCR according to the "routine-based approach". 2
Supplementary material Appendix 2: Formulas and results for imbalanced data estimation 4
Supplementary material Appendix 3: Formulas used to estimate the performances of classifiers and meta-classifiers 5

Supplementary Table S1: Mean and Standard deviation of Kappa values for each method of selection variable/sampling/Machine Learning algorithm 6
Supplementary Table S2: Performances of the meta-classifiers on the external dataset 7
Supplementary Table S3: Mean and Standard deviation of Kappa values per method on the external dataset 8
Supplemental Table S4: Comparison of each type of variable from positive versus negative classes using Wilcoxon Rank Sum Test 9
Supplementary Table S5: Number of features selected with selection variables methods (Recursive Feature Elimination) among a random loop with 50 iterations on the classifier dataset. 10
Supplementary Table S6: Parameters for training models 11

Supplementary material Appendix 1: Interpretation of the doubtful results of IDqPCR according to the "routine-based approach".

→ Table SA.1: Interpretation of the positive results from doubtful IDqPCR according to the "Routine-based approach" (17 samples)

Sexe, age	Underlying disease	Clinical symptoms	Imagery	Specimen	Direct examination/ histopathology	Culture	Treatement	EORTC-MSGRC	Evolution
M, 9*	ALL relapse	ARDS Hemorragic cerebral, digestive	Alveolar opacities	Cutaneous biopsy	Large Hyphae	Negative	AMB	**Proven**	Death
				Expectoration	Large hyphae	Negative			
				serum	NA	NA			
M, 68*	Multiple myeloma	Transplantation	Normal	Expectoration	Large hyphae	*M. circinelloides*	AMB	**Probable**	Survival
M, 64*	SOT, diabetis	Febril neutropenia	Pulmonary nodule	BALF	Large hyphae	*R. microsporus*	AMB, ISA	**Probable**	Survival
F, 63*	SOT	Febrile dyspnea	Condensation excavee	BA	Large hyphae	Negative	AMB	**Probable**	Death
M, 48*	*Legionella* pneumopathies	Multiviscerale failure	Pulmonary nodule	Digestive biopsy	Large hyphae	Negative	No antifungal treatment	**Proven**	Death
M, 38*	Pancytopenia	Dyspnea	Pulmonary nodule	Expectoration	Large and thin hyphae	*A. fumigatus*	AMB	**Probable**	Survival
F, 29*	ALL	Necrotic palatum lesion	NA	Palatum biopsy	Large hyphae	Negative	Surgery AMB, ISA	**Proven**	Survival
			NA	Serum	NA	NA			
M, 59*	ALL relapse	Hematemese Gastric liquid pos with mucorales and a fum	Cerebral abscess	Serum	NA	NA	Surgery AMB	**Proven**	Death
F, 35*	Bicytopenia, acidocetosic diabetis	Septic shock BALF with large hyphae at histopathology, and Mucor PCR positive	Excavated condensation, ground glass opacities	serum	NA	NA	AMB	**Proven**	Survival
M, 66*	SOT, severe Covid	ARDS	Non contributive	Expectoration	Negative	Negative	AMB	**NA**	Survival
				Bronchial aspiration	Negative	Negative			
M, 68*	Acidocetosic diabetis	sinusitis	Sinusitis	Biopsy	Large hyphae	Negative	Surgery, AMB, followed by ISA	**Proven**	Survival
M, 69*	MDS	Cutaneous abscess	Not done	biopsy	Large Hyphae	Negative	Surgery ISA	**Proven**	Survival

Sexe, age	Underlying disease	Clinical symptoms	Imagery	Specimen	Direct examination/ histopathology	Culture	Treatement	EORTC-MSGRC	Evolution
M, 62*	Multiple myeloma, diabtis, Severe covid	ARDS, cerebral symptoms	Pulmonary condensation, cerebral hemorrhage	serum	NA	NA	AMB	**Possible**	Death

Notes: *More than 1 specimen with mucorales PCR positive; M: male, F: Female, ALL: acute lymphoid leukemia, ARDS: acute respiratory distress syndrome, BALF: Bronchoalveolar fluid, SOT: solid organ transplant, NA: not available, BA: Bronchial aspiration, AMB: liposomal amphotericin B, ISA: isavuconazole

→ Interpretation of the negative results from doubtful IDqPCR according to the "Routine-based approach" (n = 89):

Among the 89 samples which were labelled negative, 46 had compatible defined by a radiologist imaging but other mucormycosis diagnosis was not retained:

- 21 samples from patients who survived with non-adapted treatment which was voriconazole (among which 8 invasive aspergillosis proven and 1 fusariosis proven diagnosis were retained)
- 1 sample from patient who survived with non-adapted treatment which was atovaquone (pneumocystis diagnosis was retained)
- 24 samples from patients who survived with no anti-fungal treatment (no fungal infection diagnosis was retained)

→ Details of excluded samples from doubtful IDqPCR according to the "Routine-based approach" (n=12):

Eleven samples were excluded due to insufficient clinical, biological data to render a final result:

- 12 samples from patients had compatible imagery defined by a radiologist
- 2 samples from patients who not survived with voriconazole treatment for proven aspergillosis diagnosis
- 3 samples from patients who survived with Ambisome treatment (1 proven actinomycosis was retained)
- 7 samples from patients with no diagnosis and whose outcome was not available with our means

Supplementary material Appendix 2: Formulas and results for imbalanced data estimation

The datasets were characterized with the class imbalance ratio (IR) defined as $IR = \frac{Number\ of\ instances\ in\ minority\ class\ (positives\ qPCR)}{Number\ of\ instances\ in\ majority\ class\ (negatives\ qPCR)}$ (1). The IR was at 9.8 and 8.1 for the classifier conception and the external datasets, corresponding to highly and medium imbalanced datasets, respectively.

(1) A. Sadollah and T. Sinha, *Recent Trends in Computational Intelligence*. BoD – Books on Demand, 2020.

Supplementary material Appendix 3: Formulas used to estimate the performances of classifiers and meta-classifiers

$$Kappa = \frac{Proportion\ of\ the\ observed\ agreement - Proportion\ of\ a\ random\ agreement}{1 - Proportion\ of\ a\ random\ agreement}$$

$$Accuracy = \frac{True\ Positive + True\ Negative}{True\ Negative + False\ Negative + True\ Positive + False\ Negative}$$

$$NPV = \frac{True\ Negative}{True\ Negative + False\ Negative}$$

$$PPV = \frac{True\ Positive}{True\ Positive + False\ Positive}$$

$$Sensitivity = \frac{True\ Positive}{True\ Positive + False\ Negative}$$

$$Specificity = \frac{True\ Negative}{TTrue\ Negative + False\ Negative}$$

$$F1 = \frac{2 \times precision \times recall}{precision + recall}$$

Notes : Cohen's kappa coefficient (assesses inter-rater reliability for imbalanced data and varies from −1 (total disagreement) to 0 (random classification) to 1 (total agreement)), accuracy (number of correctly predicted data), Negative Predictive Value (NPV) (proportion of the negatives cases giving negative results), Predictive Positive Value (PPV) (proportion of the positives cases giving positive results), sensitivity (true-positive recognition rate) and specificity (true-negative recognition rate), F1-score (harmonic mean of PPV and sensitivity

Supplementary Table S1: Mean and Standard deviation of Kappa values for each method of selection variable/sampling/Machine Learning algorithm

Selection variable methods	Resampling method	ML algorithm	Mean Kappa value	SD Kappa value
No_selection_var	Smote	RF	0.93	0.06
No_selection_var	Up	RF	0.92	0.06
No_selection_var	Down	RF	0.89	0.08
No_selection_var	Raw	RF	0.89	0.06
RFE_Glmnet	Raw	svm	0.89	0.07
RFE_RF	Up	svm	0.89	0.07
RFE_Glmnet	Down	nnet	0.88	0.07
RFE_Glmnet	Down	RF	0.88	0.07
RFE_Glmnet	Smote	svm	0.88	0.07
RFE_RF	Raw	svm	0.88	0.08
RFE_Glmnet	Raw	nnet	0.88	0.08
RFE_Glmnet	Raw	RF	0.88	0.08
RFE_RF	Down	svm	0.88	0.07
RFE_Glmnet	Down	svm	0.88	0.07
RFE_RF	Smote	svm	0.87	0.07
RFE_RF	Up	nnet	0.87	0.07
RFE_RF	Up	RF	0.87	0.07
RFE_RF	Raw	nnet	0.87	0.08
RFE_RF	Raw	RF	0.87	0.08
RFE_RF	Smote	nnet	0.87	0.07
RFE_RF	Smote	RF	0.87	0.07
RFE_Glmnet	Up	nnet	0.87	0.07
RFE_Glmnet	Up	RF	0.87	0.07
No_selection_var	Smote	nnet	0.87	0.07
RFE_Glmnet	Smote	nnet	0.87	0.06
RFE_Glmnet	Smote	RF	0.87	0.06
No_selection_var	Up	svm	0.87	0.08
No_selection_var	Smote	svm	0.87	0.07
RFE_Glmnet	Up	svm	0.87	0.06
RFE_RF	Down	nnet	0.87	0.08
RFE_RF	Down	RF	0.87	0.08
RFE_RF	Smote	nb	0.86	0.08
No_selection_var	Up	nnet	0.86	0.07
No_selection_var	Raw	svm	0.86	0.08
RFE_Glmnet	Smote	nb	0.86	0.08
No_selection_var	Down	nnet	0.85	0.08
No_selection_var	Smote	nb	0.85	0.08
No_selection_var	Raw	nb	0.84	0.08
No_selection_var	Raw	nnet	0.84	0.09
RFE_Glmnet	Raw	nb	0.84	0.07
No_selection_var	Down	svm	0.84	0.09
RFE_RF	Raw	nb	0.83	0.08
RFE_RF	Up	nb	0.82	0.08
No_selection_var	Up	nb	0.82	0.09
RFE_Glmnet	Up	nb	0.81	0.08
RFE_Glmnet	Down	nb	0.78	0.12
RFE_RF	Down	nb	0.76	0.14
No_selection_var	Down	nb	0.71	0.16

Notes: ML for Machine Learning, SD for Standard Deviation, NB for Naive Bayes, SVM for Linear Support Vector Machine, RF for Random Forests and nnet for single-hidden-layer Neural NETwork; Recursive Feature Elimination (RFE) coupled to Random forests (RF) or Logistic Regression (Glmnet) or No selection variable (No_selection_var) method; resampling methods (Up, Down or SMOTE (1)) or no resampling method (Raw).

(1) N. V. Chawla, K. W. Bowyer, L. O. Hall, and W. P. Kegelmeyer, 'SMOTE: Synthetic Minority Over-sampling Technique', J. Artif. Intell. Res., vol. 16, pp. 321–357, Jun. 2002, doi: 10.1613/jair.953.

Supplementary Table S2: Performances of the meta-classifiers on the external dataset

ML algorithm*	Variable selection*	Resampling*	Sensitivity	Specificity	PPV‡	NPV‡	F1‡	Accuracy‡	Kappa‡
NB	No	Down	0.992	1.000	1.000	0.936	0.996	0.993	0.963
		Raw	1.000	1.000	1.000	1.000	1.000	1.000	1.000
		SMOTE	1.000	0.977	0.997	1.000	0.999	0.998	0.987
		Up	1.000	0.977	0.997	1.000	0.999	0.998	0.987
	RFE-Glmnet	Down	1.000	1.000	1.000	1.000	1.000	1.000	1.000
		Raw	1.000	0.977	0.997	1.000	0.999	0.998	0.987
		SMOTE	1.000	1.000	1.000	1.000	1.000	1.000	1.000
		Up	1.000	0.977	0.997	1.000	0.999	0.998	0.987
	RFE-RF	Down	0.997	1.000	1.000	0.978	0.999	0.998	0.987
		Raw	1.000	0.977	0.997	1.000	0.999	0.998	0.987
		SMOTE	1.000	1.000	1.000	1.000	1.000	1.000	1.000
		Up	1.000	0.977	0.997	1.000	0.999	0.998	0.987
nnet	No	Down	0.997	1.000	1.000	0.978	0.999	0.998	0.987
		Raw	1.000	0.932	0.992	1.000	0.996	0.993	0.961
		SMOTE	1.000	0.932	0.992	1.000	0.996	0.993	0.961
		Up	1.000	0.955	0.994	1.000	0.997	0.995	0.974
	RFE-Glmnet	Down	0.997	1.000	1.000	0.978	0.999	0.998	0.987
		Raw	1.000	0.909	0.989	1.000	0.994	0.990	0.947
		SMOTE	1.000	0.909	0.989	1.000	0.994	0.990	0.947
		Up	1.000	0.909	0.989	1.000	0.994	0.990	0.947
	RFE-RF	Down	0.997	1.000	1.000	0.978	0.999	0.998	0.987
		Raw	1.000	0.909	0.989	1.000	0.994	0.990	0.947
		SMOTE	1.000	0.932	0.992	1.000	0.996	0.993	0.961
		Up	1.000	0.909	0.989	1.000	0.994	0.990	0.947
RF	No	Down	1.000	1.000	1.000	1.000	1.000	1.000	1.000
		Raw	1.000	0.773	0.973	1.000	0.986	0.975	0.858
		SMOTE	1.000	0.841	0.981	1.000	0.990	0.983	0.904
		Up	1.000	0.864	0.983	1.000	0.992	0.985	0.919
	RFE-Glmnet	Down	1.000	0.977	0.997	1.000	0.999	0.998	0.987
		Raw	1.000	0.795	0.975	1.000	0.988	0.978	0.874
		SMOTE	1.000	0.841	0.981	1.000	0.990	0.983	0.904
		Up	1.000	0.841	0.981	1.000	0.990	0.983	0.904
	RFE-RF	Down	1.000	1.000	1.000	1.000	1.000	1.000	1.000
		Raw	1.000	0.773	0.973	1.000	0.986	0.975	0.858
		SMOTE	1.000	0.841	0.981	1.000	0.990	0.983	0.904
		Up	1.000	0.841	0.981	1.000	0.990	0.983	0.904
SVM	No	Down	0.997	1.000	1.000	0.978	0.999	0.998	0.987
		Raw	1.000	0.727	0.967	1.000	0.983	0.970	0.826
		SMOTE	0.997	0.977	0.997	0.977	0.997	0.995	0.974
		Up	0.997	0.977	0.997	0.977	0.997	0.995	0.974
	RFE-Glmnet	Down	0.997	0.977	0.997	0.977	0.997	0.995	0.974
		Raw	1.000	0.841	0.981	1.000	0.990	0.983	0.904
		SMOTE	1.000	0.955	0.994	1.000	0.997	0.995	0.974
		Up	1.000	0.955	0.994	1.000	0.997	0.995	0.974
	RFE-RF	Down	1.000	0.864	0.983	1.000	0.992	0.985	0.919
		Raw	1.000	0.864	0.983	1.000	0.992	0.985	0.919
		SMOTE	1.000	0.955	0.994	1.000	0.997	0.995	0.974
		Up	1.000	0.955	0.994	1.000	0.997	0.995	0.974

Notes: *NB for Naive Bayes, SVM for Linear Support Vector Machine, RF for Random Forests and nnet for single-hidden-layer Neural NETwork; Recursive Feature Elimination (RFE) coupled to Random forests (RF) or Logistic Regression (Glmnet) or No selection variable method (No); resampling methods (Up, Down or SMOTE) or no sampling method (raw). ‡PPV: Positive Predictive Value; NPV: Negative Predictive Value; F1: mean of Sensitivity and PPV; Accuracy: agreement with reference technique (visual reading); Kappa: measure inter-rater reliability.

Supplementary Table S3: Mean and Standard deviation of Kappa values per method on the external dataset

Method	Mean Kappa value	SD Kappa value
Down	0.98	0.02
Raw	0.92	0.06
SMOTE	0.96	0.04
Up	0.96	0.03
NB	0.99	0.01
nnet	0.96	0.02
RF	0.92	0.05
SVM	0.95	0.05
No_selection_var	0.95	0.05
RFE-Glmnet	0.96	0.04
RFE-RF	0.95	0.04

Notes: SD for Standard Deviation, NB for Naive Bayes, SVM for Linear Support Vector Machine, RF for Random Forests and nnet for single-hidden-layer Neural NETwork; Recursive Feature Elimination (RFE) coupled to Random forests (RF) or Logistic Regression (Glmnet) or No selection variable (No_selection_var) method; resampling methods (Up, Down or SMOTE (1)) or no resampling method (Raw)

(1) N. V. Chawla, K. W. Bowyer, L. O. Hall, and W. P. Kegelmeyer, 'SMOTE: Synthetic Minority Over-sampling Technique', J. Artif. Intell. Res., vol. 16, pp. 321–357, Jun. 2002, doi: 10.1613/jair.953.

Supplemental Table S4: Comparison of each type of variable from positive *versus* negative classes using Wilcoxon Rank Sum Test

variable	Negative class (effective)	Positive class (effective)	*p-value-norm* (Shapiro–Wilk test)	*p-value* (Wilcoxon Rank Sum tests)
init2	660	74	< 2.2e-16	0.101
init1	660	74	< 2.2e-16	0.0454
cpD2	660	74	< 2.2e-16	6.68e-15
fluo	660	74	< 2.2e-16	8.28e-11
AUC	660	74	< 2.2e-16	0.0154
cpD1	660	74	< 2.2e-16	5.27e-39
global slope	660	74	< 2.2e-16	2.12e-43
delta fluorescence	660	74	< 2.2e-16	2.89e-25
maxRatio	660	74	< 2.2e-16	2.45e-30
AUC Tm	660	74	< 2.2e-16	9.62e-27
maximum fluorescence	660	74	< 2.2e-16	6.5e-15
Tm	660	74	< 2.2e-16	2.26e-44
kurtosis	660	74	< 2.2e-16	1.93e-44
skewness	660	74	< 4.461e-14	5.13e-45

Notes: *p-value* was adjusted with Benjamini-Hochberg procedure. A normality test (Shapiro–Wilk test) was performed (*p-value-norm*) before the Wilcoxon Rank Sum Test.

cpD1 (Cp at the first maximum derivative of the amplification curve); **cpD2** (Cp at the second maximum derivative of the amplification curve); **init1** (the initial template fluorescence from the sigmoidal model); **init2** (the initial template fluorescence from an exponential model); **fluo** (the fluorescence value the maximum of the second derivative curve (cpD2)); **maximum fluorescence** (the maximum of fluorescence of the amplification curve); **global slope** (the slope of the amplification curve using a linear regression model); **AUC amplification** (Area Under the amplification Curve); **delta fluorescence** (the difference of fluorescence between the minim and the maximum of fluorescence); **maxRatio** (this method allows the identification of a coherent point in or very close to the exponential region of the qPCR signal (1)). **Tm** (melting temperature), **AUC Tm**: area under the melting curve, **kurtosis** (measure of shape concerning the melting curve) and **skewness** (skewness, measure of asymmetry of the melting curve).

Supplementary Table S5: Number of features selected with selection variables methods (Recursive Feature Elimination) among a random loop with 50 iterations on the classifier dataset.

Variables	RFE-RF	RFE-GLMnet
AUC amplification	14	23
AUC Tm	44	34
cpD1	14	12
cpD2	14	9
delta fluorescence	26	29
fluo	10	10
global slope	48	49
init1	5	5
init2	7	2
kurtosis	42	40
maxRatio	28	23
maximum fluorescence	26	30
skewness	50	49
Tm	18	22

Notes: Recursive Feature Elimination (RFE) coupled to Random forests (RF) or Logistic Regression (Glmnet). **cpD1** (Cp at the first maximum derivative of the amplification curve); **cpD2** (Cp at the second maximum derivative of the amplification curve); **init1** (the initial template fluorescence from the sigmoidal model); **init2** (the initial template fluorescence from an exponential model); **fluo** (the fluorescence value the maximum of the second derivative curve (cpD2)); **maximum fluorescence** (the maximum of fluorescence of the amplification curve); **global slope** (the slope of the amplification curve using a linear regression model); **AUC amplification** (Area Under the amplification Curve); **delta fluorescence** (the difference of fluorescence between the minim and the maximum of fluorescence); **maxRatio** (this method allows the identification of a coherent point in or very close to the exponential region of the qPCR signal (1)). **Tm** (melting temperature), **AUC Tm**: area under the melting curve, **kurtosis** (measure of shape concerning the melting curve) and **skewness** (skewness, measure of asymmetry of the melting curve). Extraction and calculation of the features was realized whith the qPCR and PerformanceAnalytics and prcama packages (2) (3) (4).

(1) E. B. Shain and J. M. Clemens, ‘A new method for robust quantitative and qualitative analysis of real-time PCR’, Nucleic Acids Res., vol. 36, no. 14, pp. e91–e91, Aug. 2008, doi: 10.1093/nar/gkn408.

(2) C. Ritz and A.-N. Spiess, ‘qpcR: an R package for sigmoidal model selection in quantitative real-time polymerase chain reaction analysis’, Bioinformatics, vol. 24, no. 13, pp. 1549–1551, Jul. 2008, doi: 10.1093/bioinformatics/btn227.

(3) B. G. Peterson et al., PerformanceAnalytics: Econometric Tools for Performance and Risk Analysis. 2020. Accessed: Mar. 21, 2022. Available: https://CRAN.R-project.org/package=PerformanceAnalytics

(4) Borchers HW. pracma: Practical Numerical Math Functions. 2022. Available at: https://CRAN.R-project.org/package=pracma. Accessed 18 April 2022.

Supplementary Table S6: Parameters for training models

**Parameters**	**Variable selection (RFE-RF RFE-Glmnet)**	**Machine Learning algorithm**
k-fold cross validation	K = 5	K = 5
Selected variables	Minimum = 2<br>Maximum = 14	Not applicable
Metric for training	Kappa	Kappa
Grid search (for hyper-parameters estimation)	Not applicable	• RF and nnet : random<br>• NB: data.frame(fL=c(0,0.5,1.0), usekernel = TRUE, adjust=c(0,0.5,1.0))<br>• SVM: expand.grid(C = seq(0.0001, 2, length = 20))

Notes: performed with a random loop (with 50 iterations) in order to generate various classifiers using Machine Learning method coupled to several variables selection and resamples methods. Models were performed using the caret package (1). Recursive Feature Elimination (RFE) coupled to Random forests (RF) or Logistic Regression (Glmnet)

(1) M. Kuhn, ‘Building Predictive Models in R Using the caret Package’, J. Stat. Softw., vol. 28, pp. 1–26, Nov. 2008, doi: 10.18637/jss.v028.i05

Session info for R programming

```
R version 4.1.2 (2021-11-01)
Platform: x86_64-w64-mingw32/x64 (64-bit)
Running under: Windows 10 x64 (build 19042)

Matrix products: default

locale:
[1] LC_COLLATE=French_France.1252
LC_CTYPE=French_France.1252
[3] LC_MONETARY=French_France.1252 LC_NUMERIC=C
[5] LC_TIME=French_France.1252

attached base packages:
[1] parallel  stats     graphics  grDevices utils     datasets  methods
base

other attached packages:
 [1] MLeval_0.3                 qpcR_1.4-1
 [3] Matrix_1.3-4               rgl_0.108.3
 [5] minpack.lm_1.2-1           MASS_7.3-54
 [7] stringr_1.4.0              RootsExtremaInflections_1.2.1
 [9] inflection_1.3.5           doParallel_1.0.17
[11] foreach_1.5.2              iterators_1.0.14
[13] PerformanceAnalytics_2.0.4    xts_0.12.1
[15] zoo_1.8-9                  pvclust_2.2-0
[17] MALDIrppa_1.1.0            waveslim_1.8.2
[19] robustbase_0.93-9          signal_0.7-7
[21] MALDIquant_1.21            pracma_2.3.6
[23] caret_6.0-90               lattice_0.20-45
[25] ggplot2_3.3.5              writexl_1.4.0
[27] tidyr_1.2.0                dplyr_1.0.8
[29] readxl_1.3.1

loaded via a namespace (and not attached):
 [1] nlme_3.1-153        fs_1.5.2            usethis_2.1.5
 [4] lubridate_1.8.0     devtools_2.4.3      rprojroot_2.0.2
 [7] tools_4.1.2         utf8_1.2.2          R6_2.5.1
[10] rpart_4.1-15        colorspace_2.0-3    nnet_7.3-16
[13] withr_2.5.0         tidyselect_1.1.2    prettyunits_1.1.1
[16] processx_3.5.2      compiler_4.1.2      cli_3.1.1
[19] desc_1.4.0          scales_1.1.1        DEoptimR_1.0-10
[22] quadprog_1.5-8      randomForest_4.7-1   callr_3.7.0
[25] proxy_0.4-26        digest_0.6.29       pkgconfig_2.0.3
[28] htmltools_0.5.2     parallelly_1.30.0    sessioninfo_1.2.2
```

```
[31] fastmap_1.1.0        htmlwidgets_1.5.4    rlang_1.0.1
[34] rstudioapi_0.13      generics_0.1.2       jsonlite_1.8.0
[37] ModelMetrics_1.2.2.2 magrittr_2.0.2       Rcpp_1.0.8
[40] munsell_0.5.0        fansi_1.0.2          lifecycle_1.0.1
[43] stringi_1.7.6        pROC_1.18.0          brio_1.1.3
[46] pkgbuild_1.3.1       plyr_1.8.6           recipes_0.2.0
[49] grid_4.1.2           listenv_0.8.0        crayon_1.5.0
[52] splines_4.1.2        knitr_1.37           ps_1.6.0
[55] pillar_1.7.0         future.apply_1.8.1   reshape2_1.4.4
[58] codetools_0.2-18     stats4_4.1.2         pkgload_1.2.4
[61] glue_1.6.1           data.table_1.14.2    remotes_2.4.2
[64] vctrs_0.3.8          testthat_3.1.2       cellranger_1.1.0
[67] gtable_0.3.0         purrr_0.3.4          kernlab_0.9-29
[70] future_1.24.0        cachem_1.0.6         xfun_0.30
[73] gower_1.0.0          prodlim_2019.11.13   e1071_1.7-9
[76] class_7.3-19         survival_3.2-13      timeDate_3043.102
[79] tibble_3.1.6         memoise_2.0.1        hardhat_0.2.0
[82] lava_1.6.10          globals_0.14.0       ellipsis_0.3.2
[85] ipred_0.9-12
```
